# Association of Size Matching Using Predicted Heart Mass With Mortality in Heart Transplant Recipients With Obesity or High Pulmonary Vascular Resistance

**DOI:** 10.1001/jamanetworkopen.2023.19191

**Published:** 2023-06-23

**Authors:** Ran Tao, Timothy M. Hess, Adam Kuchnia, Joshua Hermsen, Farhan Raza, Ravi Dhingra

**Affiliations:** 1 Department of Medicine, University of Wisconsin-Madison, Madison; 2 Division of Cardiovascular Medicine, University of Wisconsin-Madison, Madison; 3 Department of Nutritional Sciences, College of Agricultural and Life Sciences, University of Wisconsin-Madison, Madison; 4 Division of Cardiothoracic Surgery, University of Wisconsin-Madison, Madison

## Abstract

**Importance:**

Pretransplant obesity and higher pulmonary vascular resistance (PVR) are risk factors for death after heart transplant. However, it remains unclear whether appropriate donor-to-recipient size matching using predicted heart mass (PHM) is associated with lower risk.

**Objective:**

To investigate the association of size matching using PHM with risk of death posttransplant among patients with obesity and/or higher PVR.

**Design, Setting, and Participants:**

All adult patients (>18 years) who underwent heart transplant between 2003 and 2022 with available information using the United Network for Organ Sharing cohort database. Multivariable Cox models and multivariable-adjusted spline curves were used to examine the risk of death posttransplant with PHM matching. Data were analyzed from October 2022 to March 2023.

**Exposure:**

Recipient’s body mass index (BMI) in categories (<18.0 [underweight], 18.1-24.9 [normal weight, reference], 25.0-29.9 [overweight], 30.0-34.9 [obese 1], 35-39.9 [obese 2], and ≥40.0 [obese 3]) and recipient’s pretransplant PVR in categories of less than 4 (29 061 participants), 4 to 6 (2842 participants), and more than 6 Wood units (968 participants); and less than 3 (24 950 participants), 3 to 5 (6115 participants), and 5 or more (1806 participants) Wood units.

**Main Outcome:**

All-cause death posttransplant on follow-up.

**Results:**

The mean (SD) age of the cohort of 37 712 was 52.8 (12.8) years, 27 976 (74%) were male, 25 342 were non-Hispanic White (68.0%), 7664 were Black (20.4%), and 3139 were Hispanic or Latino (8.5%). A total of 12 413 recipients (32.9%) had a normal BMI, 13 849 (36.7%) had overweight, and 10 814 (28.7%) had obesity. On follow-up (median [IQR] 5.05 [0-19.4] years), 12 785 recipients (3046 female) died. For patients with normal weight, overweight, or obese 2, receiving a PHM-undermatched heart was associated with an increased risk of death (normal weight hazard ratio [HR], 1.20; 95% CI, 1.07-1.34; overweight HR, 1.12; 95% CI, 1.02-1.23; and obese 2 HR, 1.07; 95% CI, 1.01-1.14). Moreover, patients with higher pretransplant PVR who received an undermatched heart had a higher risk of death posttransplant in multivariable-adjusted spline curves in graded fashion until appropriately matched. In contrast, risk of death among patients receiving a PHM-overmatched heart did not differ from the appropriately matched group, including in recipients with an elevated pretransplant PVR.

**Conclusion and Relevance:**

In this cohort study, undermatching donor-to-recipient size according to PHM was associated with higher posttransplant mortality, specifically in patients with normal weight, overweight, or class II obesity and in patients with elevated pretransplant PVR. Overmatching donor-to-recipient size was not associated with posttransplant survival.

## Key Points

**Question:**

Is appropriately sizing donor-to-recipient heart associated with a lower risk of posttransplant death for patients with obesity or higher pulmonary vascular resistance (PVR)?

**Findings:**

In this cohort study of 37 712 individuals, undermatching using predicted heart mass (PHM) was associated with a higher risk of death in recipients with normal weight, overweight, and class II obesity. Moreover, in patients with PVR of more than 5 Wood units, risk of death posttransplant was lower if the donor was appropriately matched close to 100% or overmatched.

**Meaning:**

These findings suggest that undersizing donor-to-recipient hearts by PHM is associated with higher posttransplant mortality specifically in patients with obesity or high PVR.

## Teaser

This cohort study evaluates the association between size matching using predicted heart mass with mortality in heart transplant recipients with obesity or high pulmonary vascular resistance.

## Introduction

Orthotopic heart transplant (OHT) is considered the reference standard treatment for patients with end-stage heart failure. With the obesity epidemic, there has been a consistent increase in body mass index (BMI; calculated as weight in kilograms divided by height in meters squared) among transplant recipients over the last few decades.^1,2,3^ Patients with obesity on the transplant list face longer wait times, with each increase in BMI associated with a 4% lower likelihood of receiving a transplant.^3,4^ Similarly, the BMIs of the available donors have also increased by 1 BMI point from 2006 to 2020.^2^

Despite the increasing prevalence of obesity among transplant donors and recipients, there is limited evidence guiding recipient selection and use of hearts from donors that are overweight or obese. Many patients die while waiting for an appropriate match on the waiting list, and many available hearts continue to be discarded.^5^ In 2006, the International Society for Heart and Lung Transplantation (ISHLT) recommended that patients with a BMI of 30 or higher should lose weight before listing for heart transplant^6^; however, the society subsequently increased their BMI threshold to 35 or higher in 2016.^7^ A recent study found recipients with BMIs between 35 and 38 may be acceptable candidates depending on individual factors and each transplant center's expertise, volume, and preference.^8^

There have been continued efforts to expand the donor pool to alleviate the long waiting time, including offering hearts from those who died from drug overdose and are hepatitis C virus positive and donation after circulatory death donors.^5^ There is no strict cutoff for donor weight requirements, as it depends on the donor-to-recipient size matching. ISHLT guidelines recommend donor body weight no greater than 30% below that of the recipient if it is a same sex transplant, or no greater than 20% for a female to male transplant.^9,10^ In 2014, Reed et al^11^ proposed predicted heart mass (PHM) calculation using donor and recipient’s height, weight, age, and sex, and found PHM matching to better predict transplant outcomes compared with BMI, especially for female donor to male recipient cases.^12^

Higher pretransplant pulmonary vascular resistance (PVR) increases the risk of posttransplant graft dysfunction and failure.^6,13^ It is recommended to confirm reversal of high pulmonary pressures using pulmonary vasodilators before proceeding to list patients for heart-alone transplant.^6,14,15^

In the present study, we examined the survival outcomes of heart transplant recipients according to BMI and PHM matching. Specifically, we focused on the outcome of transplants for those with obesity (BMI ≥30) and the association of PHM matching with obese BMI categories. Additionally, we examined the association of the recipient’s pretransplant PVR with PHM matching.

## Methods

Due to the deidentified UNOS data set and retrospective design, the need for informed consent was waived. The present study was approved by the University of Wisconsin-Madison institutional review board and followed the Strengthening the Reporting of Observational Studies in Epidemiology (STROBE) reporting guideline.

We performed a retrospective cohort study analysis of all adult heart transplants between January 1, 2003, to June 30, 2022, in the US using the United Network for Organ Sharing (UNOS) database. We excluded patients aged younger than 18 years at the time of transplantation, retransplantation, or multiorgan transplant. We also excluded patients with missing data, specifically for recipient height, recipient weight, donor height, donor weight, or those with incomplete follow-up. Information on race and ethnicity were directly extracted from the UNOS database. Participants were categorized as non-Hispanic White, Black, Hispanic, Asian, or other (other included Indian American or Pacific Islander and Native Hawaiian or Alaska native categories). Race and ethnicity were assessed because prior investigations have reported disparities in care and clinical outcomes among minorities.^16^

Patients were divided into 6 categories according to their pretransplant BMI: underweight (<18), normal weight (18.0-24.9), overweight (25.0-29.9), obese class I (30.0-34.9), obese class II (35.0-39.9), and obese class III (≥40). In the present study, we a priori analyzed the association of PHM matching on survival posttransplant among patients with obesity in different categories of BMI.

Predicted heart mass was calculated according to the formula defined by Reed et al^11^:

- Predicted left ventricular mass: *a* × height^0.54^ × weight^0.61^; a = 6.82 for women and *a* = 8.25 for men
- Predicted right ventricular mass: *a* × age^−0.32^ × height^1.135^ × weight^0.315^; *a* = 10.59 for women and a = 11.25 for men
- PHM = predicted left ventricular mass + predicted right ventricular mass

We considered an OHT to be appropriately matched when the PHM ratio, defined as Donor PHM / Recipient PHM × 100%, was within 86% to 114%. Undermatch and overmatch were defined as PHM ratio less than 86% and greater than 114%, respectively.

### Statistical Analysis

Baseline characteristics of patients were compared according to BMI categories as described previously. Continuous measures were presented as medians with IQRs and compared using the Kruskal-Wallis test, while binary and categorical variables were presented as counts with percentages and compared using the χ^2^ test. We first examined the observed time to death by generating Kaplan-Meier curves according to BMI categories. This was replicated within the undermatched, matched, and overmatched PHM subgroups. The log-rank test was used to compare the rates of survival by BMI categories.

Next, we constructed multivariable Cox regression models to evaluate the risk of death after covariate adjustment with full sample and separately within each BMI category according to PHM matching where the matched category served as a referent. We constructed all multivariable models in a stepwise fashion by including all covariates from Table 1 (except for BMI and PHM matching score) and using a .10 significance level to variable retention, and a .05 significance level for possible reentry into the model. Age and sex were forced into the model at all stages. Then, we created spline curves^17^ examining the risk of death according to donor-recipient PHM matching, as unadjusted, multivariable-adjusted full cohort, and multivariable-adjusted with separate BMI categories.

**Table 1.** Baseline Characteristics of Heart Transplant Recipients According to Body Mass Index Status

Characteristics	Participants, median (IQR)						
	Underweight (n = 636)	Normal (n = 12 413)	Overweight (n = 13 849)	Obese I (n = 8299)	Obese II (n = 2277)	Obese III (n = 238)	*P* value^a^
Recipient age, y	44 (27-58)	56 (44-63)	57 (48-63)	55 (47-61)	52 (43-59)	47 (38-55)	<.001
Recipient height, cm	168 (160.0-177.8)	172.7 (165.1-180.3)	175.3 (167.6-180.34)	175.3 (167.6-180.3)	172.7 (165.1-180.3)	172.7 (162.6-179.0)	<.001
Recipient weight, kg	48.1 (43.5-54.0)	67.3 (59.6-74.1)	83.9 (76.7-90.7)	98.4 (90.3-106.2)	110.8 (101.0-120.0)	122.5 (111.3-133.4)	<.001
Recipient PHM^b^	125.2 (109.4-150.5)	165.0 (134.3-180.2)	189.6 (170.5-202.8)	208.0 (184.4-222.6)	221.4 (184.5-238.9)	231.7 (186.3-251.1)	<.001
Sex, No. (%)							
Female	306 (48)	3863 (31)	2899 (21)	1933 (23)	647 (28)	88 (37%)	<.001
Male	330 (52)	8550 (69)	10 950 (79)	6366 (77)	1630 (72)	150 (63%)	
Donor age, y	28 (19-39)	29 (21-40)	30 (22-41)	31 (23-41)	32 (24-41)	34 (24-42)	<.001
Donor PHM	156.5 (132.8-182.2)	173.6 (147.9-193.1)	186.7 (165.9-205.3)	197.1 (176.9-216.9)	201.8 (180.9-222.2)	205.7 (181.6-226.6)	<.001
Recipient race and ethnicity, No. (%)							
Non-Hispanic White	352 (55)	8193 (66)	9620 (69)	5655 (68)	1399 (61)	123 (51)	<.001
Black	144 (23)	2344 (19)	2584 (19)	1833 (22)	671 (29)	88 (37)	
Hispanic	56 (9)	1109 (9)	1188 (9)	609 (8)	156 (7)	21 (9)	
Asian	77 (12)	664 (5)	331 (2)	104 (1)	19 (1)	2 (1)	
Other^c^	7 (1)	112 (1)	126 (1)	98 (1)	32 (1)	4 (2)	
Recipient education, No. (%)							
High school	217 (34)	4213 (34)	5081 (37)	3263 (39)	1006 (44)	105 (44)	<.001
College	318 (50)	6545 (53)	6986 (50)	4161 (50)	1027 (45)	97 (41)	
Serum creatinine, mg/dL^d^	1.0 (0.8-1.2)	1.1 (0.9-1.4)	1.2 (1.0-1.5)	1.2 (1.0-1.5)	1.2 (1.0-1.5)	1.2 (0.9-1.5)	<.001
Total bilirubin, mg/dL^e^	0.8 (0.5-1.4)	0.8 (0.5-1.3)	0.7 (0.5-1.2)	0.7 (0.5-1.1)	0.7 (0.4-1.0)	0.8 (0.5-1.2)	<.001
Life support, No. (%)							
Intravenous inotropes	338 (53)	5606 (45)	5343 (39)	2719 (33)	654 (29)	66 (28)	<.001
Mechanical ventilator	18 (3)	262 (2)	275 (2)	128 (2)	38 (2)	7 (3)	.02
IABP	79 (12)	1351 (11)	1287 (9)	717 (9)	158 (7)	13 (5)	<.001
VAD	95 (15)	2395 (19)	3406 (25)	2482 (30)	816 (36)	87 (37)	<.001
ECMO	16 (3)	198 (2)	194 (1)	108 (1)	34 (1)	4 (2)	.14
HLA mismatch	5 (4.0-5.5)	5 (4-5.5)	5 (4-5.5)	5 (4-5.5)	5 (4-6)	5 (4-6)	.09
cPRA >10%	111 (17)	1865 (15)	1899 (14)	1276 (15)	382 (17)	43 (18)	<.001
Ischemic time, h	3.3 (2.6-3.9)	3.2 (2.5-3.9)	3.2 (2.5-3.8)	3.3 (2.5-3.9)	3.3 (2.5-3.9)	3.3 (2.6-4.0)	<.001
Time on waitlist, d	39 (12-133)	52 (16-161)	89 (24-260)	123 (34-345)	150 (37-395)	105 (32.5-305.5)	<.001
Sex match							
Male donor to female recipient	112 (18)	2084 (17)	2075 (15)	800 (10)	193 (8)	21 (9)	<.001
Female donor to male recipient	116 (18)	1521 (12)	1234 (9)	862 (10)	306 (13)	44 (18)	
PHM match							
Under match	22 (3)	1121 (9)	1949 (14)	1507 (18)	624 (27)	72 (30)	<.001
Over match	391 (61)	4427 (36)	2711 (20)	1142 (14)	255 (11)	22 (9)	

Abbreviations: cPRA, calculated panel reactive antibody; ECMO, extracorporeal membrane oxygenation; HLA, human leukocyte antigens; IABP, intra-aortic balloon pump; PHM, predicted heart mass; VAD, ventricular assist device.

^a^ *P* values for continuous variables are from the Kruskal-Wallis test while binary and categorical measure comparisons used the χ^2^ test.

^b^ Undermatch is defined as less than 86% and overmatch as more than 114% of PHM ratio between donor and recipient.

^c^ Race and ethnicity data were directly extracted from the UNOS database. The “other” category included Indian American or Pacific Islander and Native Hawaiian or Alaska Native categories.

^d^ To convert creatinine to µmol/L, multiply by 76.25.

^e^ To convert bilirubin to µmol/L, multiply by 17.104.

To further elucidate the association of recipient’s PVR with PHM matching and recipient’s survival posttransplant, we first examined the interaction between PVR and PHM matching. Because we found a statistically significant interaction, we further created multivariable-adjusted spline curves accounting for all donor and recipient factors associated with risk to examine the PVR in categories of less than 4, 4 to 6, and more than 6 Wood units (WU, a common measure of PVR)^13^ as well as in categories of less than 3, 3 to 5, and more than 5 WU^18^ to examine the risk of death posttransplant against PHM in a continuous fashion. Two separate categorical models were created because recommendations for PVR cut-offs have evolved over time specifically for pulmonary hypertension diagnosis although recommendations for heart transplant eligibility criteria have not consistently changed.

A *P* value of less than .05 was considered statistically significant. All analyses were performed using SAS software version 9.4 (SAS Institute). All graphics were produced using R version 4.2.1 (R Project for Statistical Computing). Data were analyzed from October 2022 to March 2023.

## Results

### Baseline Characteristics

Pretransplant demographics of the recipients are displayed in Table 1, according to BMI categories. A total of 47 613 heart transplant recipients were extracted from the UNOS database. Among them, 9439 were excluded according to the exclusion criteria, and an additional 462 were excluded due to missing data. A total of 37 712 patients were included in the final analysis data set (eFigure in Supplement 1). The mean (SD) age was 52.8 (12.8) years, and 9736 (26%) were female. The most common race and ethnicity for transplant recipients was non-Hispanic White (25 342 participants [68.0%]), followed by Black (7664 participants [20.4%]), Hispanic or Latino (3139 participants [8.5%]), and Asian (1197 participants [3.2%]) (Table 1).

In the overall cohort, mean (SD) BMI of recipients was 27.3 (4.9), in which 12 413 (32.9%) were normal weight, 13 849 (36.7%) overweight, and 10 814 (28.6%) were obese. Among obese recipients, the majority (22.0%) had class I obesity (BMI 30.0-34.9), 6.0% had class II obesity (BMI 35.0-39.9), and 0.6% had class III obesity (BMI ≥40). In addition, 1.7% of recipients had a BMI of 18.0 or less and were categorized as underweight.

Recipients who were either underweight or had class II or III obesity were younger compared with other categories. The percentage of patients receiving ventricular assist device support increased with increasing BMI categories. In contrast, patients were overall less likely to receive intravenous inotropes or intra-aortic balloon pump support at the time of transplant with higher BMI categories. Extracorporeal membrane oxygenation use was similar between different categories.

Patients in higher BMI categories, specifically with a BMI of 30 or higher had a longer wait time on the waiting list. Recipients with higher BMIs were less likely to receive a PHM-oversized donation and more likely to receive a PHM-undersized donation. Those with a higher BMI in general were also more likely to receive sex-matched donations.

### Transplant Outcomes According to PHM-Matching

**Figure 1.**
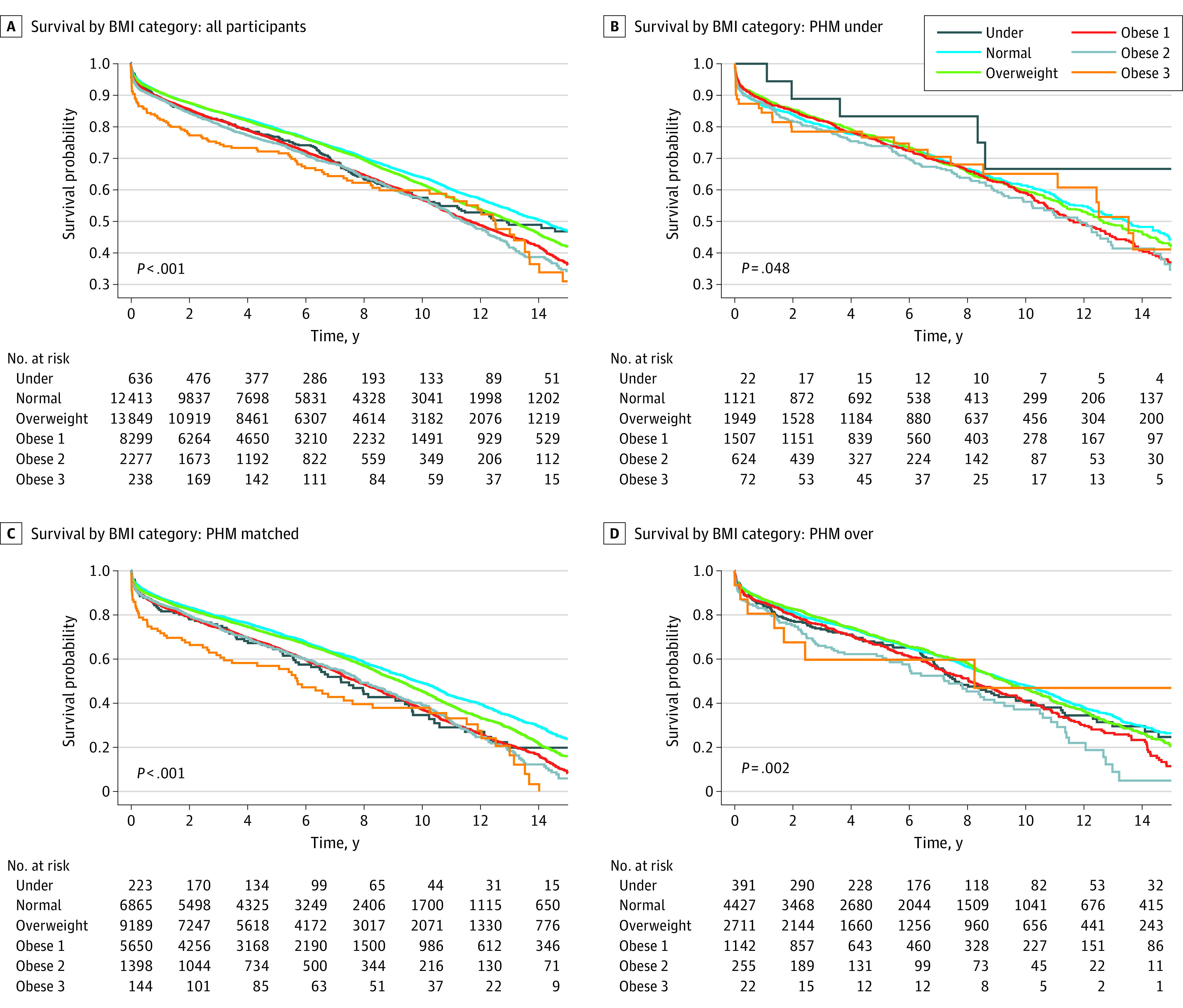
Kaplan-Meier Curves According to Body Mass Index (BMI) and Predicted Heart Mass (PHM) Matching Strategy Kaplan-Meier curves for each PHM matching strategy according to recipient’s baseline BMI status. Kaplan-Meier curves of all patients (panel A), undermatched patients (panel B), matched patients (panel C), and overmatched patients (panel D). The *P* values presented are from the log-rank test comparing the 6 survival curves.

Upon follow-up (median [IQR] 5.05 [0-19.4] years), 12 785 recipients died. Person-years of follow-up were calculated for the whole cohort and according to PHM matching (eTable in Supplement 1). The unadjusted Kaplan-Meier curves showed a significant difference in survival rates posttransplant in different BMI categories, specifically for the obese class III category compared with other groups (log rank *P* <. 0001; χ^2^_5_ = 123.9) (Figure 1A). Furthermore, survival rates on follow-up remained significantly different for patients in BMI categories when the cohort was separately examined in subgroups of PHM undermatched, adequately matched, and overmatched (Figure 1B, C, and D) (log rank *P* < .05 for all; undermatched: log rank *P *= .048; χ^2^_5_ = 11.2; adequately matched: log rank *P* < .001; χ^2^_5_ = 102.3; and overmatched: log rank *P* = .002; χ^2^_5_ = 18.6) and visually, the survival appeared to be markedly lower in the obese class III group compared with other categories, specifically in PHM matched subgroup.

In multivariable Cox models, when patients were examined according to matching and according to BMI categories, recipients with normal weight, overweight or class II obesity had about 7% to 20% higher risk of death with PHM undermatching compared with appropriately matched recipients (Table 2). Risk of death was similar in PHM overmatched groups compared with appropriately matched recipients in all BMI categories (Table 2). For patients with normal weight, overweight, or obese 2, receiving a PHM-undermatched heart was associated with an increased risk of death (normal weight hazard ratio [HR], 1.20; 95% CI, 1.07-1.34; overweight HR, 1.12; 95% CI, 1.02-1.23; and obese 2 HR, 1.07; 95% CI, 1.01-1.14).

**Table 2.** Multivariable Cox Regression Models Examining the Risk of Death According to BMI Categories in Individual PHM Matched Subgroups^a^

BMI and PHM^b^	No.	Hazard ratio (95% CI)	*P* value
Entire cohort (n = 37 712)			
Undermatched	5295	1.02 (0.96-1.08)	.59
Matched	23 469	1 [Reference]	NA
Overmatched	8948	1.01 (0.96-1.06)	.71
Underweight recipients (n = 636)			
Undermatched	22	0.57 (0.21-1.59)	.28
Matched	223	1 [Reference]	NA
Overmatched	391	0.74 (0.52-1.04)	.08
Normal weight recipients (n = 12 413)			
Undermatched	1121	1.20 (1.07-1.34)	.001
Matched	6865	1 [Reference]	NA
Overmatched	4427	1.08 (0.99-1.18)	.07
Overweight recipients (n = 13 849)			
Undermatched	1949	1.12 (1.02-1.23)	.01
Matched	9189	1 [Reference]	NA
Overmatched	2711	0.99 (0.91-1.09)	.90
Obese class I recipients (n = 8299)			
Undermatched	1507	0.96 (0.85-1.07)	.42
Matched	5650	1 [Reference]	NA
Overmatched	1142	0.91 (0.80-1.03)	.15
Obese class II recipients (n = 2277)			
Undermatched	624	1.07 (1.01-1.14)	.02
Matched	1398	1 [Reference]	NA
Overmatched	255	0.96 (0.91-1.01)	.12
Obese class III recipients (n = 238)			
Undermatched	72	0.72 (0.42-1.24)	.23
Matched	144	1 [Reference]	NA
Overmatched	22	0.76 (0.32-1.80)	.54

Abbreviations: NA, not applicable; PHM, predicted heart mass.

^a^ We used stepwise modeling, and all variables as noted in Table 1 were included if they remained significant at *P* < .10 for all multivariable Cox regression models. Covariates that remained in the final model included age, sex (forced into the model) and total bilirubin, calculated panel reactive antibody greater than 10%, human leukocyte antigens mismatch, glomerular filtration rate category, ischemic time, ventilator at transplant, extracorporeal membrane oxygenation at transplant, and intra-aortic balloon pump at transplant. This final adjusted model included 34 814 patients. For the instances where predicted heart mass category hazard ratios were significant, the proportional hazard assumption was tested with the Kolmogorov supremum test and no association was found.

^b^ Undermatched is defined as <86% and overmatched as >114% of PHM ratio between donor and recipient.

**Figure 2.**
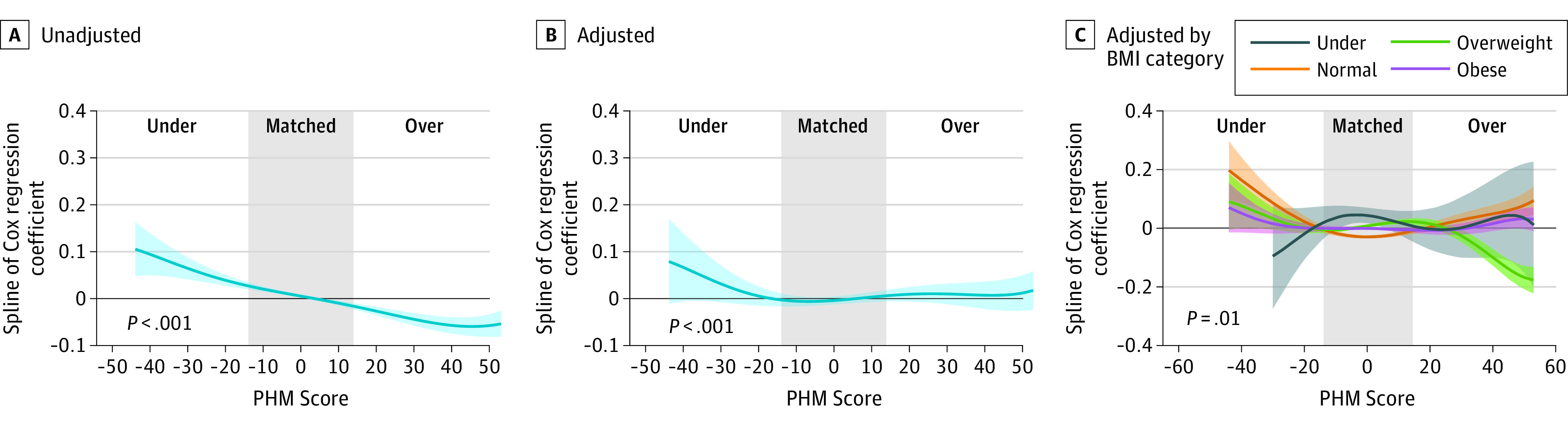
Spline Curves According to Predicted Heart Mass (PHM) Matching Strategy Spline analysis of mortality, according to different PHM matching. Panel A is unadjusted, and panel B is adjusted for Cox regression covariates. Panel C is adjusted for Cox regression covariates and controlled by baseline BMI. The *P* values in panels A and B test whether the PHM score is associated with mortality, while the *P* value in panel C is for the test of whether the association of PHM with mortality differs between BMI categories.

Because we used cut-offs based on PHM matching to examine these associations, we studied these associations in a more continuous fashion using spline analysis against PHM scores. We obtained similar results when we performed the spline analysis examining the risk of death in unadjusted (Figure 2A), multivariable-adjusted (Figure 2B), and multivariable models stratified by BMI (Figure 2C). The risk of death was higher for undermatched transplant and approached null with matching in adjusted curves and remained null with overmatching (*P* < .001; χ^2^_3_ = 136.1) (Figure 2B) in multivariable-adjusted spline model. Moreover, when stratified according to BMI categories, the risk of death was higher within each BMI category for undermatched individuals, remained lowered for appropriately matched, but became slightly higher again for normal weight or obese individuals when overmatched by 30% or more (Figure 2C).

### PHM Matching According to Pretransplant PVR Categories

**Figure 3.**
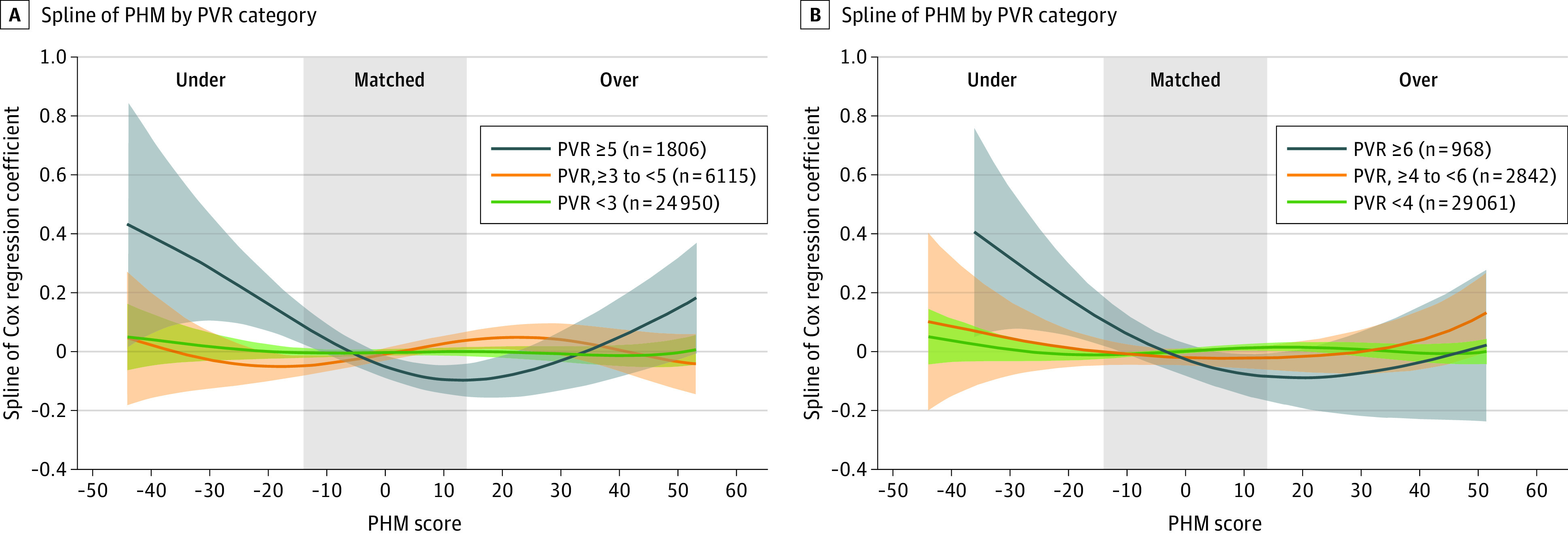
Spline Curves by Pulmonary Vascular Resistance (PVR) Categories and Predicted Heart Mass (PHM) Matching Strategy Spline analysis of mortality, according to pretransplant PVR that are categorized as less than 3, 3 to 5, and more than 5 Wood units (panel A), and as less than 4, 4 to 6, and more than 6 Wood units (panel B).

Because of significant difference in association of PHM matching groups when examining the risk of death by PVR classification, PVR stratified multivariable-adjusted spline curves were created. Spline analysis examining the risk of death stratified by pretransplant PVR of less than 3, 3 to 5, and more than 5 WU did show higher risk of death within the group with PVR more than 5 WU specifically with PHM undermatching such that the risk of death was higher until matching reached almost 100% (Figure 3A). The risk of death remained lower in all 3 categories of PVR with overmatching. Similarly, when PVR categories were created according to less than 4, 4 to 6, and more than 6 WU, there was a graded association observed with higher risk of death with undermatching among patients with PVR 4 to 6 or more than 6 WU up to the point when PHM matching reached 100% (Figure 3B) and then continued to essentially remain lower.

## Discussion

### Principal Findings

Our study confirmed the results from previous studies that for recipients with BMI between 18.0 and 24.9, receiving a PHM-undermatched heart was associated with increased risk. Contrary to common belief, transplanting an overmatched heart was generally not associated with a worse outcome for recipients except for some BMI categories with more than 130% matching. Additionally, for recipients with an elevated pretransplant PVR, receiving a PHM undermatched heart was associated with an increased posttransplant risk of death. In fact, spline curves displayed a higher risk of death posttransplant with PHM undermatching until matching with donor reached up to 100% of recipient PHM. Risk remained lower on overmatching for all groups of PVR, whereas there was clearly a graded association with PHM-matching, with higher risk of death associated with higher PVR.

### Comparison With Literature

A previous study by Kransdorf et al^19^ examined the outcomes of OHT recipients between 2007 and 2016 according to septiles of donor-recipient PHM and reported that receiving a severely undersized donor heart (defined as a PHM ratio <0.863) was associated with increased early mortality posttransplant. We saw the same outcome with the updated and more contemporary UNOS database; specifically for normal weight recipients (BMI 18.0-24.9), receiving an undermatched heart was associated with a 20% increased risk of death posttransplant. Similarly, overweight or patients with class II obesity also had a significantly higher risk of death if they received an undermatched heart, although the risk may have been slightly lower. These associations did not achieve statistical significance in the extreme BMI (<18.0 and ≥40.0) categories because of relatively smaller sample. However, it may also be associated with other factors, such as the obesity paradox, termed after the findings that obesity may be associated with lower mortality in patients with advanced heart failure, although the exact mechanism remains elusive.^20,21,22^ Therefore, we further provided supporting evidence that is in agreement with the current ISHLT recommendation,^15^ that undermatched heart transplant should be avoided. Specifically, transplanting a heart that is undermatched per PHM criteria (<86%) for recipients with higher baseline PVR should be avoided.

In our study, overmatching was not associated with an increased posttransplant mortality risk. The same finding was also seen in the study by Kransdorf et al.^19^ We further categorized the recipients according to different BMI categories and found no BMI groups had an increased risk of death after receiving a heart considered as overmatched. As described by Shah et al,^23^ overmatched OHT was not associated with an increased risk, especially for those with an elevated pretransplant PVR, which was also confirmed in our study. Additionally, in the present study, we observed the association between PVR and survival to be affected by appropriately matching donors to recipients. Patients with higher PVR may benefit from matching close to 100% of donor PHM to reduce posttransplant death risk.

### Potential Mechanisms

Obesity is a major risk factor for heart failure and other cardiovascular diseases. Morbid obesity is a known independent risk factor for developing heart failure, with a reported BMI increase of 1 being associated with a 5% to 7% increased risk of developing heart failure.^24^ Changes in hemodynamics, including higher metabolic demand and changes in total blood volume and stroke volume, can increase the stress on the heart, leading to structural changes and earlier development of heart failure. The reverse has been reported in patients undergoing bariatric surgery. Weight loss postbariatric surgery was associated with improvement in both ejection fraction and subjective improvement in functional capacity.^25^ It is increasingly difficult for patients with end-stage heart failure to lose weight (besides considering bariatric surgery) and experts only recommend 5% to 10% weight loss before considering advanced therapies.^26^ In fact, the Association of the European Society of Cardiology has recently removed recommendations for actively pursuing weight loss in patients with severe chronic heart failure due to risk of becoming sarcopenic (sarcopenic obesity) or cachexic.^27^

Pretransplant obesity also increases periprocedural risks related to anesthesia, longer cardiopulmonary bypass time, prolonged ventilator support, higher risk of postsurgical infection, wound dehiscence, and thrombosis.^28,29^ Posttransplant, recipients with obesity, especially those with class II and class III obesity, have been found to have an increased risk of postsurgical hypertension, diabetes, kidney dysfunction, malignant neoplasms, stroke, acute or chronic rejections, CAV, and infection, compared with normal weight counterparts.^30,31^

Heart failure–associated pulmonary hypertension leads to pulmonary vascular remodeling that is more pronounced in venous vasculature^32^ and is associated with greater exercise-induced lung congestion, right ventricular–pulmonary arterial uncoupling, impairment in oxygen delivery, and impaired aerobic capacity.^33^ It is possible that patients who underwent transplant with high PVR have combined precapillary and postcapillary pulmonary hypertension. Unfortunately, the UNOS database does not capture information on vasoreactivity testing that every center performs for patients with high PVR at baseline to further characterize patients with different types of pulmonary hypertension. Nonetheless, in our study, recipients with higher PVR had a lower risk of death posttransplant with appropriate or higher PHM matching.

### Strengths and Limitations

Evaluation of a large national database, long-term follow-up and appropriate adjustment for confounders in multivariable models are strengths of our study. Our study also has some limitations. First, it is an observational cohort study, so we cannot attribute causal relations from these results. Even though many potential confounding factors that influence posttransplant death risk were accounted for in the model, there are possibilities such as surgical complications or infections, which could not be accounted for in this analysis. However, these occasional instances are random and bias us toward null. We also did not account for hospital volume as some centers that maybe are more aggressive than others in accepting match offers are out of PHM matching range. However, we submit that in the past 10 years, hospital volumes have substantially changed due to a national increase in heart transplant rates that leads to misclassification of transplant centers as low, medium, or high-volume centers. Examining accurate weight in heart failure patients is also challenging because many advanced heart failure patients tend to retain fluid, which makes it difficult to ascertain a true calculation of BMI for a patient. However, most programs list these patients for transplant knowing that size is important in matching donors, therefore any true differences in weight due to fluid gain are perhaps also random and again bias us toward null. Additionally, it is recommended to perform vasodilatory challenge to assess reversibility in patients with high PVR before transplant. Unfortunately, the UNOS database did not collect information that would allow us to conduct additional analysis.

## Conclusions

In our cohort study, pretransplant obesity was a factor associated with risk for death posttransplant. Appropriately matching donor hearts according to PHM was associated with a lower posttransplant mortality risk, whereas undermatching was associated with an increased risk. Additionally, for recipients with elevated pretransplant PVR (>5 WU) matching closer to 100% or higher of donor PHM was associated with lower posttransplant mortality risk.

## Notes

Supplement 1. **eFigure. **Consort Diagram of Study Data Acquisition From the United Network for Organ Sharing Database **eTable. **Mortality According to Predicted Heart Mass (PHM) Matching

Supplement 2.
Data Sharing Statement
