# Supplementary material for: Association of Size Matching Using Predicted Heart Mass With Mortality in Heart Transplant Recipients With Obesity or High Pulmonary Vascular Resistance
Source: JAMA Netw Open. 2023 Jun 23;6(6):e2319191. doi: 10.1001/jamanetworkopen.2023.19191 (PMC10290246; doi:10.1001/jamanetworkopen.2023.19191)
Supplement: Supplement 1. — eFigure. Consort Diagram of Study Data Acquisition From the United Network for Organ Sharing Database eTable. Mortality According to Predicted Heart Mass (PHM) Matching [file jamanetwopen-e2319191-s001.pdf]

Supplemental Online Content

Tao R, Hess TM, Kuchnia A, Hermsen J, Raza F, Dhingra R. Association of size matching using predicted heart mass with mortality in heart transplant recipients with obesity or high pulmonary vascular resistance. *JAMA Netw Open*. 2023;6(6):e2319191. doi:10.1001/jamanetworkopen.2023.19191

eFigure. Consort Diagram of Study Data Acquisition From the United Network for Organ Sharing Database
eTable. Mortality According to Predicted Heart Mass (PHM) Matching

This supplemental material has been provided by the authors to give readers additional information about their work.

© 2023 Tao R et al. *JAMA Network Open.*

eFigure. Consort Diagram of Study Data Acquisition From the United Network for Organ Sharing Database

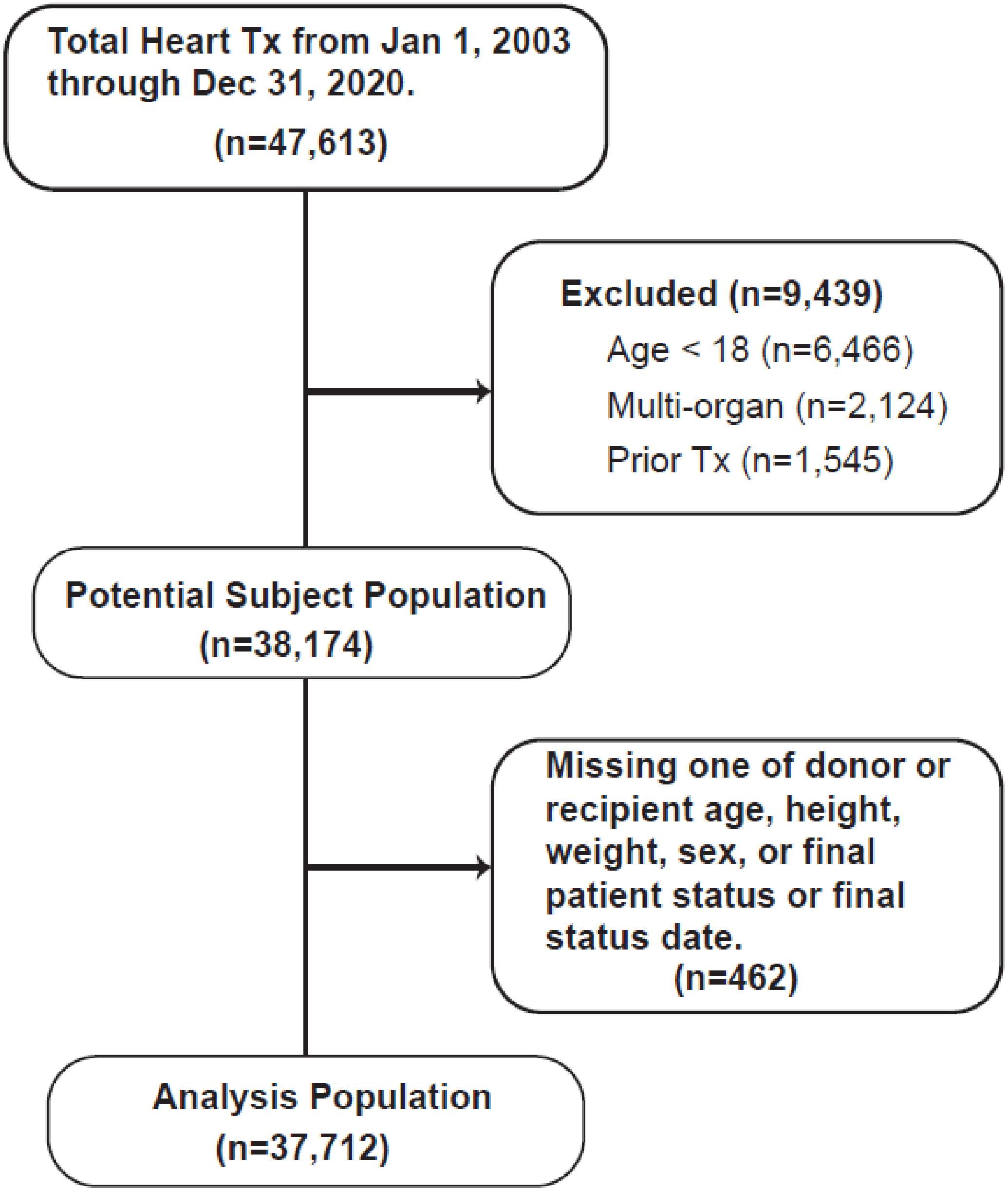

© 2023 Tao R et al. *JAMA Network Open.*

eTable. Mortality According to Predicted Heart Mass (PHM) Matching

PHM	Number of Transplants	Died	% died	Person-years of Follow up	Median Survival Time (95% Conf Int)
All Patients	37,712	12,785	33.9%	6.2	13.01 (12.77 – 13.24)
Under matched	5,295	1,915	36.2%	6.2	12.57 (12.12 – 13.06)
Matched	23,469	7,913	33.8%	6.2	12.93 (12.63 – 13.19)
Over matched	8,948	2,939	32.9%	6.3	13.62 (13.10 – 14.17)

© 2023 Tao R et al. *JAMA Network Open.*
