# Supplementary material for: Association of Size Matching Using Predicted Heart Mass With Mortality in Heart Transplant Recipients With Obesity or High Pulmonary Vascular Resistance
Source: JAMA Netw Open. 2023 Jun 23;6(6):e2319191. doi: 10.1001/jamanetworkopen.2023.19191 (PMC10290246; doi:10.1001/jamanetworkopen.2023.19191)
Supplement: Supplement 2. — Data Sharing Statement [file jamanetwopen-e2319191-s002.pdf]

# Data Sharing Statement

Tao. Association of Size Matching Using Predicted Heart Mass with Mortality in Heart Transplant Recipients With Obesity or High Pulmonary Vascular Resistance. *JAMA Netw Open*. Published June 23, 2023. doi:10.1001/jamanetworkopen.2023.19191

**Data**
**Data available:** No

**Additional Information**
**Explanation for why data not available:** Already available publicly through UNOS by request
